# Insect farming: A bioeconomy-based opportunity to revalorize plastic wastes

**DOI:** 10.1016/j.ese.2024.100521

**Published:** 2024-12-24

**Authors:** Juan C. Sanchez-Hernandez, Mallavarapu Megharaj

**Affiliations:** a Laboratory of Ecotoxicology, Institute of Environmental Sciences, University of Castilla-La Mancha, 45071, Toledo, Spain; b Global Centre for Environmental Remediation (GCER), College of Engineering, Science and Environment, University of Newcastle, Callaghan, NSW, 2308, Australia

**Keywords:** Insect-based bioconversion, Plastic waste, Micro/nanoplastics, Biochar, Bioeconomy

## Abstract

Managing plastic waste is one of the greatest challenges humanity faces in the coming years. Current strategies—landfilling, incineration, and recycling—remain insufficient or pose significant environmental concerns, failing to address the growing volume of plastic residues discharged into the environment. Recently, increasing attention has focused on the potential of certain insect larvae species to chew, consume, and partially biodegrade synthetic polymers such as polystyrene and polyethylene, offering novel biotechnological opportunities for plastic waste management. However, insect-assisted plastic depolymerization is incomplete, leaving significant amounts of microplastics in the frass (or manure), limiting its use as a soil amendment. In this perspective, we propose a novel two-step bioconversion system to overcome these limitations, using insects to sustainably manage plastic waste while revalorizing its by-products (frass). The first step involves pyrolyzing microplastic-containing frass from mealworms (*Tenebrio molitor* larvae) fed on plastic-rich diets to produce biochar with enhanced adsorptive properties. The second stage integrates this biochar into the entomocomposting of organic residues, such as food waste, using black soldier fly (*Hermetia illucens*) larvae to produce nutrient-rich substrates enriched with carbon and nitrogen. This integrated system offers a potential framework for large-scale industrial applications, contributing to the bioeconomy by addressing both plastic waste and organic residue management. We critically examine the advantages and limitations of the proposed system based on current literature on biochar technology and entomocomposting. Key challenges and research opportunities are identified, particularly concerning the physiological and toxicological processes involved, to guide future efforts aimed at ensuring the scalability and sustainability of this innovative approach.

## Highlights

• A novel two-step bioconversion system for managing plastic and food waste streams.
• Biochar is proposed as the core of the system, improving entomocomposting of food waste.
• Toxicology evaluation of microplastics and biochar ensures system safety.

## Graphical abstract

**Figure:**
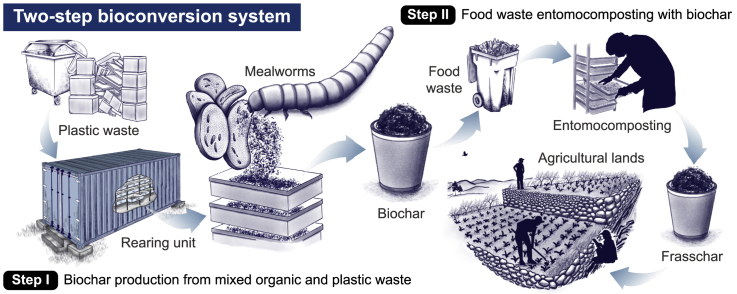

## 1 Introduction

The exponential growth of the global human population in the coming years demands an expansion of livestock systems, which could have significant environmental consequences, including deforestation, biodiversity loss, land degradation, increased greenhouse gas (GHG) emissions, higher water consumption, greater reliance on external energy inputs, and the generation of environmentally harmful residues [1]. Alternative protein sources are urgently needed to address these pressing issues. Insect farming has gained attention as a sustainable and efficient protein production system that could alleviate the environmental pressures associated with conventional livestock systems [2,3]. The primary application of insect farming is feed production [4]. Many insect species are rich in protein, polyunsaturated fatty acids, minerals, and essential amino acids [5,6], and some of them exhibit an efficient feed conversion rate [7]. Another important application of insect rearing is converting organic waste into valuable by-products [8]. Insect species such as the black soldier fly (*Hermetia illucens*) can utilize a wide variety of organic waste as food, including food waste, fermented maize straw, palm kernel expeller, soybean curd residues, brewer’s spent grain, mushroom stems, animal manure, and biosolids [[10], [11], [12], [13], [14], [9]]. The common housefly (*Musca domestica*) can thrive on wheat bran, food waste, and manure [9,10,12,14], while the house cricket (*Acheta domesticus*) can consume brewery waste, fruits, vegetables, laying hen feed, and by-products of the wine industry [9,10,14]. Coleopteran species such as the yellow mealworm (*Tenebrio molitor*) and superworm (*Zophobas atratus*) predominantly feed on agricultural by-products (e.g., wheat bran), as well as fresh inedible plant biomass [9,10,14].

Insect farming also benefits agriculture by producing frass, a manure-like residue composed of a mixture of larval feces, undigested food, fragments of dead larvae, and larval exuviae [7,15]. Its nutrient levels are generally higher than common organic fertilizers such as poultry litter [16]. The nitrogen, phosphorus, and potassium contents in commercial insect frass typically are 1.6–6.97%, 0.7–8.7%, and 0.7–8.1%, respectively [7], depending on the insect species and feedstock type [17]. Numerous studies have demonstrated insect frass promotes plant growth and health [15,18]. Nutrient supply, chitin content, labile organic matter content, and the presence of plant growth-promoting bacteria and fungi are the primary factors driving the positive effects of frass on plant development [19]. For example, composted frass produced by *H. illucens* larvae fed on brewer’s spent grain significantly increased maize growth when applied at a rate of 2.8 tons ha^−1^, equivalent to 100 kg nitrogen ha^−1^ [20]. Similarly, the frass of *T. molitor* fed with wheat bran increased barley biomass at 10 tons of dry mass ha^−1^ [21]. The biomass, flowering, and growth rate of 13 plant species grown in mealworm frass-treated soils also significantly increased in most cases at a dose of 0.5% (v/v) compared to unfertilized controls, or they were similar to plants grown in soil amended with 0.3% (v/v) hen manure [22]. Likewise, frass exhibits protective effects against plant pathogens [9,15]. For instance, the frass of *H. illucens* and *A. domesticus*, applied at rates of 10 and 5 g kg^−1^ soil, respectively, effectively controlled the plant pest *Delia radicum* (cabbage root fly) [23].

**Fig. 1:**
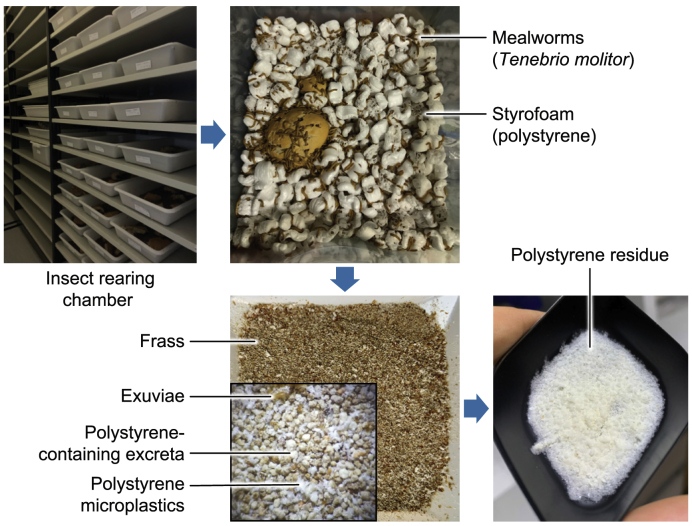
Use of mealworms (*Tenebrio molitor* larvae) for managing polystyrene plastic waste. In this example, mealworms were reared for a month on a diet of 10% polystyrene mixed with spent bread. The resulting frass contained a substantial amount of microplastics, extracted by treating the frass with H_2_O_2_ following the method described by Hurley et al. [135].

In recent years, there has been increasing interest in the ability of certain coleopteran (*T. molitor* and *Z. atratus*) and lepidopteran (*Galleria mellonella*) larvae to consume and potentially biodegrade plastic polymers, including polyethylene, polystyrene, polypropylene, polyurethane, and polyvinyl chloride [[24], [25], [26], [27], [28]]. This metabolic capability, assisted by microbial gut symbionts [26,29,30], offers an exciting opportunity to address one of the most pressing environmental challenges—plastic pollution. However, the insect-assisted biodegradation of plastics remains incomplete [[31], [32], [33]], leaving significant amounts of microplastics in the frass and raising concerns about its safe and sustainable application as a soil amendment (Fig. 1). Addressing these limitations and identifying innovative strategies for managing these by-products are crucial to advancing this emerging field.

To explore potential solutions, we systematically reviewed the peer-reviewed literature on the use of insects in plastic waste management. This process followed the methodological framework of the Preferred Reporting Items for Systematic reviews and Meta-Analyses (PRISMA, eligibility criteria in Supplementary Material Fig. S1). We conducted a bibliometric analysis using VOSviewer software [34] on 215 studies to identify research trends, knowledge gaps, key insect species, and the types of plastic polymers studied. The results revealed an exponential increase in research on this topic over the last five years, establishing it as an emerging research area, with notable contributions from Asian countries (China, India, and South Korea) and the United States (Supplementary Material Fig. S2A). The analysis also showed that polyethylene and polystyrene are the most studied plastic polymers, primarily in coleopteran and lepidopteran insect groups (Supplementary Material Fig. S2B). While plastic biodegradation remains the dominant focus of current research, the study of plastic toxicity remains comparatively underexplored, as previously highlighted [35]. Moreover, given the significant role of gut symbionts in facilitating plastic biodegradation, there is growing interest in isolating these microorganisms for biotechnological applications targeting plastic breakdown (Supplementary Material Fig. S2A). Despite this progress, no studies have addressed the disposal or revalorization of frass, larvae, and pupae of insects intentionally exposed to plastic-rich diets, highlighting a critical gap in bio-based plastic management.

This perspective aims to bridge these gaps by proposing a novel two-step bioconversion system incorporating pyrolysis and insect-based processes to manage plastic waste while revalorizing its by-products sustainably. In the first step, the microplastic-containing frass is subjected to pyrolysis to solve plastic contamination and produce upgraded biochar. In the second step, this biochar is used as a co-composting agent with food waste to produce a carbon- and nitrogen-enriched substrate, termed “frasschar,” distinguishing it from biochar-free frass. Considering the known environmental benefits of biochar and its established role as a bulking agent in conventional composting of organic residues, we explore its potential utility in entomocomposting. Our approach is focused on two insect species—mealworm and black soldier fly—because of their established role in organic waste bioconversion, extensive knowledge of their biology compared to other insects used for similar purposes [4,19,36], ease and low cost of rearing [5,10], and the diverse environmental and industrial applications of their by-products [10,15,16,21,37]. The two hypothetical stages outlined here are based on the principle of ecotechnology and offer potential applications for mitigating the environmental pressures associated with two globally problematic waste streams: plastic and food waste. While the proposed bioconversion system is nearing practical applicability, further research is needed to optimize its efficiency and minimize potential environmental risks. To this end, we have identified several knowledge gaps that must be addressed to advance the development of sustainable insect-based waste management systems.

## 2 Biochar produced from blended organic and plastic waste streams

Landfilling, incineration, and recycling are currently the main strategies for plastic waste management [38]. While recycling is the most desirable option, it accounts for only 9% of the approximately 6300 Mt of plastic waste produced since 1950 [39]. One alternative to mitigate the environmental impact of plastic waste is the development of biodegradable polymers, which can reduce the accumulation of non-biodegradable plastics in the environment [40]. However, fluctuating environmental variables, including temperature and moisture, may limit the biodegradability of these polymers observed under laboratory conditions, raising concerns about their actual biodegradation rates in the environment [41,42]. Another promising option is the pyrolysis of pure plastic waste, which generates high-grade biofuels [43,44]. However, this process faces technical challenges, such as forming corrosive and high-viscosity by-products and reducing the reactor’s operational lifespan [[45], [46], [47]].

Pyrolysis is the thermochemical decomposition of organic materials (e.g., biomass) at elevated temperatures (300–700 °C) in an oxygen-limited atmosphere. It involves covalent bond dissociation, free radical formation, aromatic condensation, and molecular rearrangement, and ends with three main by-products: syngas (gaseous fraction), bio-oils (liquid fraction), and biochar (solid fraction) [46]. Syngas and bio-oils are primarily used for energy generation and chemical production [48], whereas biochar is generally used as a soil amendment. Its high porosity, large surface area, and environmental stability make biochar an effective adsorbent material for removing pollutants from water and soil [49]. Additionally, biochar indirectly supports plant development and health by altering soil physicochemical and biological properties, such as increased water retention, nutrient availability, and the activity and composition of microbial communities [50]. Biochar also reduces GHG emissions from soil [51] and during composting processes [52], making it a valuable tool for combating global climate change [53]. Furthermore, biochar is an excellent physical support for immobilizing and stabilizing enzymes of industrial and environmental relevance [54,55]. However, these benefits are highly dependent on the physicochemical properties of biochar, which are determined by pyrolysis conditions (e.g., temperature and duration) and feedstock characteristics [56]. Accordingly, biochar may exhibit potential adverse effects on soil function because of intrinsic toxic compounds formed during pyrolysis and interactions of biochar with existing soil contaminants [57,58]. Therefore, physicochemical biochar characterization and a predictive environmental risk assessment are critical to identifying potential adverse effects, particularly when plastics are included as co-feedstock in the pyrolysis process.

Several studies have demonstrated the potential of pyrolysis when applied to blended organic and plastic residues, resulting in biochar with enhanced adsorptive properties. For example, the co-pyrolysis of corn stover and polystyrene or polyethylene terephthalate originated biochars with larger surface areas and pore size than biochars from corn stover alone [59]. The pyrolysis of plastic bags or agricultural mulch films mixed with biomass produced biochar rich in carboxylate anions, amides, and aromatic groups on its surface [60]. Similarly, the pyrolysis of spent agricultural mulch films and biomass (poultry litter or swine manure) generated biochar with an improved capacity to bind soil extracellular enzymes, likely due to the plastic-induced formation of carbonyl groups on the biochar surface [55]. Furthermore, the co-pyrolysis of spent polyethylene terephthalate bottles with rice straw produced biochar with enhanced pollutant immobilization capabilities compared to biochar produced from rice straw [61]. One challenge in this co-pyrolysis strategy is achieving a homogeneous mixture of organic and plastic residues, with plastic content representing at least 50% of the mix, to optimize synergistic effects [45]. Pre-treatment methods, such as heating [62] or cryo milling [63], can increase the density of plastic residues, facilitating their homogenization with biomass before pyrolysis. An alternative approach involves using insect larvae capable of chewing plastic waste.

Mealworms can fragment polystyrene foam into particles that are homogeneously mixed with frass (Fig. 1), potentially offering a cost-effective and technically more straightforward solution for mixing biomass and plastics than mechanical methods. The ability of coleopterans to chew plastic materials has been well-documented since World War II, driven by the need to preserve packaged food from insect infestation during its storage and transport to areas of the conflict [64]. Studies in the early 1950s revealed that many insect species could chew polyethylene film, a synthetic polymer gaining popularity at the time [65]. This observation was confirmed six decades later when wax moth larvae *G. mellonella* were found to chew polyethylene plastic bags [66], partially degrading the polymer, possibly due to specific salivary enzymes [67]. In their natural environment, mealworms are soil-dwellers with significant burrowing activity [68], which likely explains the intense drilling observed when they feed expanded polystyrene foam (Fig. 1). Although these insects exhibit high chewing activity toward plastic waste, complete biodegradation of the plastic is not achieved, leaving a significant amount of microplastics in the frass [24,69,70]. This residual microplastic content discourages the use of frass as an organic fertilizer.

As previously discussed, the pyrolysis of plastic-containing frass could offer a viable solution for revalorizing this residue. Some studies have demonstrated that pyrolysis of insect frass generates biochar suitable for pollutant removal. For example, Fe-activated biochar from mealworm frass removed up to 98.3% of malachite green dye in solution [71]. Non-activated biochar produced from mealworm frass at 800 °C for 90 min was also highly efficient at removing this toxic dye from water (1738 mg g^−1^ biochar), with higher adsorption capacities than other types of biochar produced with different feedstocks [72]. Similarly, biochar from the frass of mealworms fed with different feedstocks (wheat bran, wheat straw, rice straw, rice bran, rice husk, and corn straw) displayed higher metal adsorption capacities than biochars made from original feedstocks [73]. Another study found that biochar produced from *H. illucens* frass pyrolyzed at 450 °C was among the most effective for Cd^2+^ adsorption (42–55 mg Cd^2+^ g^−1^ biochar) compared to 14 other biochars from different feedstocks [74]. Neonicotinoids were also efficiently removed (155–325 mg g^−1^ biochar) from pesticide-spiked solutions with KOH-activated biochar produced from mealworm frass at 500 °C for 1 h [75]. Interestingly, the same biochar type displayed high CO_2_ adsorption (3.05 mol kg^−1^) and exhibited electrochemical properties [76].

**Fig. 2:**
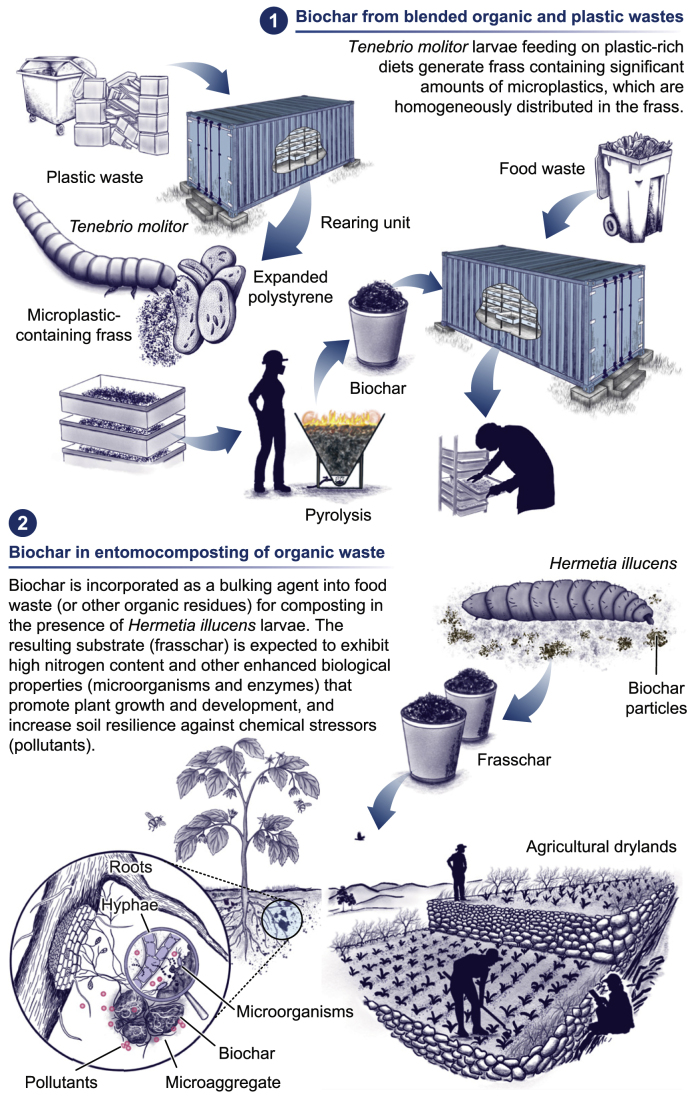
Schematic representation of a two-step bioconversion system for plastic waste management. The first step employs larvae of coleopteran larvae (*Tenebrio molitor*) to produce microplastic-rich frass, which undergoes pyrolysis to generate biochar. In the second step, this biochar is used as an additive in entomocomposting organic residues, such as food waste, utilizing dipteran larvae (*Hermetia illucens*). The final product, referred to as “frasschar,” is expected to exhibit enhanced properties (e.g., nutrients, microorganisms, enzymes) compared to biochar-free frass, contributing to improve soil fertility and resilience against adverse environmental conditions. Rearing units illustrated are adapted from designs by Entocycle (U.K.) and Manna Insect (Finland). Illustration by Juan C. Sanchez-Hernandez.

Beyond its application in contaminated water remediation, biochar derived from *H. illucens* frass suggests significant agronomic potential due to its high nitrogen content. For example, biochar from *H. illucens* frass produced at 500 °C contained 3.9 ± 0.1% nitrogen, more than double the nitrogen content of biochars derived from anaerobic digestate (1.9 ± 0.1%) and sewage sludge (1.4 ± 0.9%) [77]. Similarly, frass-derived biochar obtained from pyrolysis at 450 and 750 °C had a nitrogen content level of 4.14 ± 0.02% and 2.60 ± 0.07%, respectively. These values exceed the nitrogen content reported for biochars produced from diverse feedstocks, including livestock manures, forestry residues, woody biomass, or spent growing media [78]. The high nitrogen content of frass-derived biochar is probably attributed to chitin, a nitrogen-rich polymer composed of N-acetylglucosamine units [79]. Insect frass contains larval exuviae; in the case of *H. illucens*, these exuviae contain 10–11% chitin [80,81]. This could explain the nitrogen-enriched biochar derived from larval frass. Additionally, the chemical composition of the feedstock significantly influences the elemental composition of the resulting biochar, as observed in studies involving mealworms fed on diverse agricultural waste [72,73]. These studies suggest that frass-derived biochar holds significant potential as a dual-purpose material, combining pollutant-removal capabilities with nutrient-release properties. Whether the presence of microplastics in frass further enhances these beneficial characteristics of its biochar remains an open question that warrants future research. We propose a hypothetical two-step bioconversion strategy to explore this possibility to transform plastic residues into biochar with enhanced remediation and fertilization properties (Fig. 2).

The biochar produced in the first step of plastic biotransformation could be suitable as a soil amendment, provided it meets regulatory requirements for toxic residues. For example, the mean concentrations of 16 polycyclic aromatic hydrocarbons in *H. illucens* frass-derived biochar produced at 450 and 600 °C were below the standard limit of the European Biochar Certificate (6 ± 2.4 mg kg^−1^ dry mass), qualifying it as “class Agro” biochar for soil fertilization [78]. Nevertheless, the pollutant concentration in biochar depends on feedstock and pyrolysis conditions, including temperature, residence time, and type of pyrolytic kiln [82,83]. Optimizing these technical parameters is, therefore, essential to producing biochar that is compliant with current soil amendment regulations. In contrast, the last larval stage of mealworms, generally used for feed and food production [2–5], is unsuitable for such purposes when larvae are rearing with plastic-rich diets because of the risk of micro- and nanoplastic accumulation in their tissues. Nevertheless, the protein, lipid, and chitin contents of these larvae can be exploited in industrial applications such as bioplastics, cosmetics, lubricants, biodiesel, and bioactive compounds, including antioxidants and antimicrobial peptides [6,10,37,84,85].

A major challenge in biochar production is its economic cost. While its environmental and agronomic benefits are widely recognized, several barriers hinder its widespread adoption, particularly in small-scale rural farming systems [86]. Biomass pyrolysis often requires sophisticated systems that rely on external fuel or electricity to achieve and maintain the high temperatures necessary for pyrolysis. This dependence on external energy, combined with logistical challenges, such as feedstock transportation, homogenization, and drying, has been identified in life cycle assessment (LCA) studies as a significant limitation [87,88]. Energy-efficient pyrolysis systems are being explored to address these technical and economic barriers. Solar pyrolysis, for instance, shows promise but is highly dependent on local solar irradiation and involves complex configurations that significantly increase costs at an industrial scale [89,90]. In contrast, flame curtain pyrolysis systems, such as conical deep-cone bowls or Kon-Tiki kilns, offer a more practical alternative. These systems are simpler to operate [91], suitable for low-income settings [92], and emit fewer uncondensed gases [[93], [94], [95]]. Furthermore, LCA studies indicate that flame curtain kilns have lower environmental impacts than other biochar-pyrolysis systems [96]. Integrating a flame curtain pyrolytic unit near a mobile insect-rearing facility (Fig. 2) could provide a cost-effective and sustainable solution, enhancing the economic viability of biochar production while reducing the carbon footprint of the bioconversion process.

In our bioconversion system, biochar derived from microplastic-containing frass could also be used as an additive in entomocomposting food waste and other organic residues. In this process, *H. illucens* larvae play a central role. This novel approach offers several advantages, addressing the challenges of composting wet organic residues, such as food waste, which include risks of anaerobiosis, excessive GHG emissions, and pathogen proliferation.

## 3 Biochar as a bulking material in the entomocomposting of organic waste

Entomocomposting, which utilizes insects to decompose solid organic waste, has become an effective waste management strategy [97]. Among the various insect species, the larva of the black soldier fly (*H. illucens*) stands out as a versatile option for breaking down a wide range of organic residues, including food waste, animal manure, biosolids, and agricultural residues [4,10,11]. However, the composition of the feedstock and the larval density can influence the quality of the resulting frass, which often requires an additional composting phase to ensure full maturation [9,98]. Furthermore, organic residues such as animal manure and biosolids may contain environmental pollutants that can compromise the decomposition process and affect the quality of both frass and larvae for subsequent biotransformation applications (e.g., feed production, cosmetics, or biofuel). As a result, food waste emerges as the most suitable organic feedstock for rearing *H. illucens* in combination with biochar. Nevertheless, there are regulatory restrictions concerning the use of food waste to rear this species for feed and food production purposes [4]. Managing this organic waste is particularly relevant in modern society [99]. For instance, in the European Union, approximately 88 million tons of food waste are generated annually, costing around 143 billion euros [100]. Although composting and anaerobic digestion are viable methods for managing food waste [101,102], they face limitations such as high facility costs, difficulties in disposing of by-products (e.g., anaerobic digestate), and GHG emissions. The voracious appetite of *H. illucens* larvae for food waste offers a complementary waste conversion system with several advantages, such as significant feedstock reduction, pathogen removal, shorter transformation times, and lower GHG emissions compared to traditional composting methods [103]. However, the high moisture content of food waste can inhibit aerobic decomposition by larvae, leading to undesirable anaerobic degradation. Incorporating biochar as a bulking agent can mitigate these risks.

The benefits of biochar as an additive in composting and vermicomposting processes have been extensively documented in the literature [52,[104], [105], [106], [107]]. Among its main effects, biochar generally enhances substrate aeration and prevents clumping while increasing pH and cation exchange capacity [104,106]. Additionally, biochar accelerates the decomposition of organic matter, increasing microbial abundance and enzyme activity within the substrate [104]. It also promotes the formation of humic substances and nitrogen mineralization [52]. Furthermore, biochar reduces nutrient loss during the composting of organic matter, mitigates GHG emissions and odor [108], and contributes to immobilizing potentially toxic elements, thereby decreasing their bioavailability [52,105,106].

Despite these established benefits, knowledge regarding the impact of biochar addition in entomocomposting remains limited, with only three studies having explored this strategy to date. One study found that larvae of *H. illucens* fed a modified Gainesville fly diet (comprising a mix of cornmeal, wheat bran, lucerne, and sugar beet pulp) containing 4% (w/w, dry mass) of sewage sludge-derived biochar exhibited no adverse effects on larval growth [109]. Moreover, the larvae showed reduced bioaccumulation of metals compared to those reared on a biochar-free diet, suggesting that metal immobilization was facilitated by biochar. A second study demonstrated that the addition of corn straw biochar at doses of 2%, 5%, and 8% (w/w) to the *H. illucens*-assisted bioconversion of soybean dregs—a viscous organic waste generated during soymilk production—resulted in decreased emissions of NH_3_ and N_2_O while increasing the total nitrogen content in the frass [110]. Additionally, larvae in biochar-treated substrates gained significantly more weight during the first five days of bioconversion than controls (biochar-free substrate), and their survival rates were higher in the biochar-amended groups. In another study, the entomocomposting of brewery’s spent grains, amended with rice husk-derived biochar at concentrations of 5%, 10%, 15%, and 20% (w/w, dry mass), revealed that biochar addition increased the nitrogen and potassium content of the resulting frass and significantly shortened the maturation time [97]. These findings suggest that incorporating biochar into entomocomposting with *H. illucens* larvae can accelerate the bioconversion process and improve the fertility potential of the resultant frass.

In our proposed two-step bioconversion model, we anticipate that these benefits could be further enhanced by utilizing upgraded biochar derived from microplastic-containing frass. However, similar to the challenge faced by *T. molitor* larvae reared on plastic-rich diets, *H. illucens* larvae and pupae may not be suitable for feed or food production due to the presence of biochar in the feedstock and regulatory restrictions on using food waste for rearing *H. illucens*, at least in certain regions as the European Union [10]. Under these constraints, directing the larvae toward industrial or pharmacological applications may be more appropriate. For instance, recent advancements have indicated the potential for producing chemically and physically stable protein-derived bioplastics from *H. illucens* larvae and pupae reared on the organic fraction of municipal solid waste [111].

## 4 Research gaps and opportunities

**Fig. 3:**
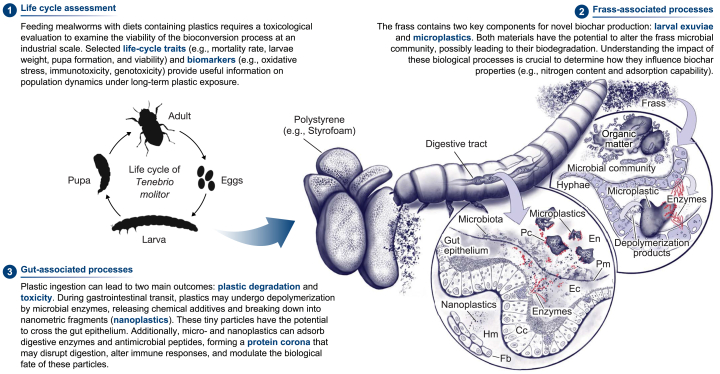
Key research priorities in plastic bioconversion using mealworms, focusing on life-cycle assessment and gut- and frass-associated processes. For further details, refer to Section 4. Cc: columnar cells; Ec: ectoperitrophic space; En: endoperitrophic space; Hm: hemocoel; Fb: fat body; Pc: protein corona; Pm: peritrophic matrix. Illustration by Juan C. Sanchez-Hernandez.

**Fig. 4:**
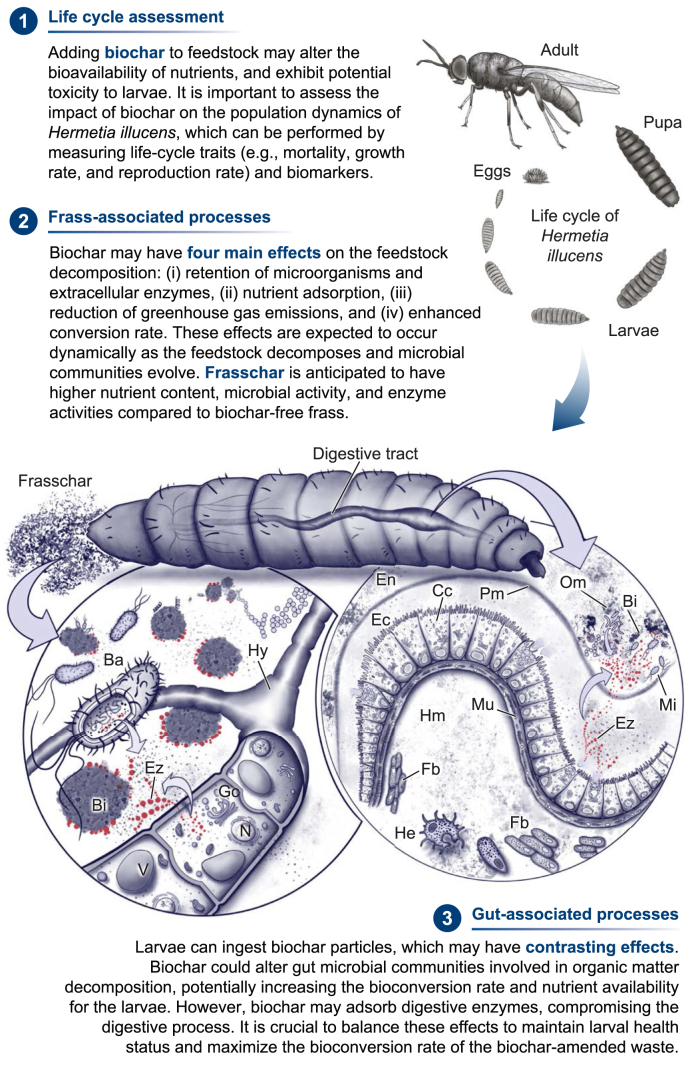
Key research priorities in entomocomposting biochar-amended organic residues using black soldier fly (Hermetia illucens) larvae. For further details, refer to Section 4. Ba: bacteria, Bi: biochar; Cc: columnar cell; Ec: ectoperitrophic space; En: endoperitrophic space; Ez: enzymes; Fb: fat body; Gc: Golgi complex; He: hemocytes; Hm: hemocoel; Hy: fungal hyphae, Mi: microorganisms; Mu: muscular layer; N: nucleus; Om: organic matter; Pm: peritrophic matrix; V: vacuole. Illustration by Juan C. Sanchez-Hernandez.

Two physicochemical stressors play an important role in the dynamic of our insect-based bioconversion system: microplastics and biochar. Although numerous studies provide valuable insights into the potential of mealworms to biodegrade plastic polymers, the toxicity of these synthetic materials in insect larvae remains limited [35]. Similarly, while much has been learned about the impact of incorporating biochar in conventional composting and vermicomposting of organic waste, little is known about the interactions between this carbonaceous material and insect larvae. Therefore, we underscore several knowledge gaps related to the exposure of insect larvae to microplastics and biochar in the following sections, considering their impact on the dynamics of the larval population in the system and their influence on gut- and frass-associated processes. Fig. 3, Fig. 4 illustrate the major physiological processes that could be compromised by microplastics and biochar, providing key messages for future research avenues.

### 4.1 Assessment of larval life-cycle traits

Optimizing the health and population dynamics of mealworms is essential for producing microplastic-rich frass suitable for pyrolysis [112]. Although mealworms exhibit a high capacity to consume plastics [28], it is important to recognize that plastics do not provide a nutritious food source. Diets composed entirely of plastics lead to high mortality rates and significant reductions in larval growth. However, supplementing plastic diets with natural feed (e.g., wheat bran and vegetables) mitigates these adverse effects [35]. Moreover, assessing the multigenerational effects of plastic bioconversion is critical for its potential industrial-scale, long-term application. For example, *T. molitor* larvae exposed to microplastics derived from polyethylene and poly(butylene adipate-coterephthalate)-starch blend mulch films across two successive generations showed no adverse effects on larval development and growth [113]. However, reduced molting was observed in the first generation exposed to higher concentrations (5% w/w) of polyethylene microplastics. Similar mitigation of microplastic-induced adverse effects over successive generations has also been documented in mealworms fed polyethylene plastic waste [69]. This phenomenon suggests some degree of adaptation to plastic-rich diets across generations. Further research is needed to explore potential plastic-induced toxicity and elucidate the mechanisms driving this adaptation. Such knowledge could advance our understanding of microplastic toxicity and the biodegradation potential of larvae adapted to plastic-rich diets [35].

Another critical consideration is the industrial scalability of plastic bioconversion using mealworms. Most studies addressing this topic have been conducted at a laboratory scale, where plastic fragments are provided alone or mixed with natural mealworm feed. Specific plastic consumption is recorded over a defined period and expressed as milligrams of plastic consumed daily per 100 larvae [114]. While consumption rates do not necessarily equate to complete ingestion and biodegradation of plastic in the larval gastrointestinal tract, they offer a useful estimate of mealworms' capacity to fragment large plastic pieces into smaller particles, which subsequently appear in the frass (Fig. 1). Laboratory-scale studies have shown that mealworms exhibit an average polystyrene consumption rate of 0.2–0.7 g of plastic per 100 larvae per day when plastic fragments are mixed 1:1 (w/w) with wheat bran [69,[115], [116], [117]]. These data could provide a foundation for scaling up the bioconversion system.

Concerning biochar, its addition to entomocomposting by *H. illucens* appears to have no adverse effects on larval growth [109], even at concentrations as high as 20% (w/w, dry mass) [97]. However, this novel approach requires future research to explore how biochar can enhance high-quality frass production while maintaining high larval yields. Investigating the potential benefits and trade-offs of biochar addition in frass production is an encouraging area for future research.

### 4.2 Gut-associated processes

The ingestion of plastic fragments by larvae induces toxicity through several mechanisms, including physical damage to the gastrointestinal epithelium, the release of potentially harmful plastic additives (e.g., phthalate esters, polybrominated flame retardants, or bisphenol A), and toxicity from monomers and oligomers (e.g., styrene) formed during polymer breakdown [118,119]. Consequently, toxicological endpoints associated with microplastic exposure typically include gut histopathology, oxidative stress biomarkers, immunotoxicity, genotoxicity, gut dysbiosis, and alterations in biomolecules and metabolites, often investigated using “omic” technologies [120]. Despite substantial efforts to understand the biodegradation capacity of mealworms, knowledge of plastic toxicity in insect larvae remains limited [121,122].

Fig. 3 presents a conceptual model of microplastic and nanoplastic distribution in mealworms and their interactions with potential biological targets. Continuous ingestion of plastic fragments may lead to multiple adverse effects in the gastrointestinal tract, including epithelial abrasion, destruction of the peritrophic matrix, disruption in nutrient digestion and absorption, and alterations in gut symbiont communities [35]. Furthermore, studies with non-insect species have shown that the gastrointestinal transit of microplastics can generate nano-sized fragments or nanoplastics (<1 μm). For example, a higher proportion of low-density polyethylene, polylactic acid, and polybutylene adipate terephthalate microplastics (20–113 μm in size) was observed in the gastrointestinal tract of *Lumbricus terrestris* compared to the surrounding soil, suggesting that this earthworm species physically breaks down ingested microplastics [123]. Similarly, Antarctic krill (*Euphausia superba*) fragmented polyethylene microbeads (31.5 ± 7.6 μm, mean ± standard deviation) included in their algal diet into particles of 7.1 ± 6.2 and 6.0 ± 5.0 μm, which were found in their digestive system and fecal material, respectively [124]. Crickets (*Gryllodes sigillatus*) also demonstrated the ability to reduce polyethylene microplastics (100 μm) to particles smaller than 1 μm, likely facilitated by the grinding action of their cuticular spines and plates lining the foregut [125].

The presence of nanoplastics in the gut lumen may have two direct toxicological consequences for larvae: (1) translocation to internal tissues and organs and (2) sequestration of biomolecules such as digestive enzymes, resulting in the formation of a protein corona—a layer of proteins and other biomolecules adsorbed onto the nanoparticle surface upon exposure to biological fluids [126]. Research on aquatic organisms indicates that nanoplastics can cross the gut epithelium and invade internal tissues, with some particles even being absorbed by cells [127]. For example, in Antarctic krill, particles of 150–500 nm in size were found in the digestive gland, indicating translocation from the gut [124]. Likewise, nanoplastics in the gut lumen can interact with digestive enzymes, forming a protein corona, which may reduce enzyme availability and alter nanoplastic toxicity [128,129]. Understanding these interactions is essential for assessing the potential adverse effects of nanoplastics in mealworms, especially under extreme microplastic exposure conditions encountered in plastic bioconversion processes.

As discussed, incorporating biochar into composting and vermicomposting offers multiple benefits for the process and the final products. However, no studies have examined the impact of ingested biochar particles on the digestive function of *H. illucens*. Unlike mealworms, *H. illucens* larvae are generalist detritivores that absorb semisolid material rather than chewing their food. This implies that biochar particle ingestion depends on the compatibility of particle size with the larval mouth opening. Although no studies have specifically investigated biochar consumption by this species, some research suggests that these larvae can ingest solid particles, such as microplastics, smaller than their mouth opening size (<110 μm) [130,131]. Assuming *H. illucens* larvae can ingest biochar particles below this size limit, Fig. 4 illustrates the hypothetical effects that biochar may have on the digestive process. Biochar could alter gut symbiont communities and enzyme activities, as observed in other invertebrates such as earthworms [132]. Additionally, biochar may adsorb various components of the larval digestive system, including nutrients, enzymes, antimicrobial peptides, and microorganisms, potentially compromising digestion and immune responses. However, these sorptive processes mean that biochar could be enzymatically and microbiologically functionalized during gut transit, resulting in biologically active frasschar that enhances fertilizer quality. These speculative processes require further research to assess the impact of biochar addition in *H. illucens*-assisted composting.

### 4.3 Frass-associated processes

Insect frass has been identified as a source of multiple bioactive compounds, including proteins, peptides, humic substances, and phytohormones, with beneficial effects on soil-plant systems [15,103]. In our bioconversion system, the frass derived from mealworms fed on plastic-rich diets contains two key components: larval exuviae, which serve as a significant source of nitrogen, and microplastics, which function as an additive in frass pyrolysis (Fig. 3). However, both components could influence the decomposition dynamics of the frass through their interactions. Specifically, the chitin in larval exuviae could promote the growth of chitinolytic microorganisms that convert chitin into chitosan [7]. Likewise, the microplastic content of the frass could be colonized by microorganisms, including chitinolytics, capable of depolymerizing these particles using various enzymes, such as peroxidases, esterases, lipases, cutinases, and chitinases [133,134]. This metabolic feedback creates a unique substrate for future research to discover potential plastic degraders. Similarly, frasschar produced from *H. illucens* feeding on biochar-amended food waste may serve as another intriguing source of bioactive compounds, owing to the interactions between biochar, gut symbionts, microorganisms, and their secreted compounds (Fig. 4).

## 5 Conclusions

Efforts to manage plastic and food waste streams play a pivotal role in mitigating environmental pollution and open new avenues for developing revalorized by-products within the bioeconomy framework. In this context, we propose a two-stage bioconversion strategy to address these environmentally challenging residues, generating organic substrates (e.g., frasschar) with potentially enhanced properties that promote soil health and plant growth. The insect-based system integrates two materials—microplastics and biochar—whose toxicity to system functionality and the environmental risks associated with their by-products necessitate comprehensive evaluation. This bio-based approach provides a valuable opportunity to explore the mechanisms of toxic action associated with microplastics and biochar particles in insects. Furthermore, the gut- and frass-associated processes triggered by microplastic exposure could serve as niches for isolating and characterizing potential microbial microplastic degraders. Biochar, recognized by the Intergovernmental Panel on Climate Change as an effective strategy for CO_2_ removal, also exhibit excellent qualities as a soil amendment and pollution remediator. However, research on its biological effects remains relatively limited compared to the extensive chemical and physical insights gained during biochar technology development. The proposed insect-based system offers a unique platform to use *H. illucens* as a model organism for investigating biochar-induced effects across various levels of biological organization, from digestive and immune functions to developmental and transgenerational impacts. We believe that the insect-based bioconversion of plastic and food waste streams represents an innovative approach with significant potential for both industrial and environmental applications. Within this framework, microplastics and biochar establish a domain for generating new scientific knowledge while contributing to sustainable waste management practices.

## CRediT authorship contribution statement

**Juan C. Sanchez-Hernandez:** Writing - Review & Editing, Writing - Original Draft, Validation, Conceptualization. **Mallavarapu Megharaj:** Writing - Review & Editing, Writing - Original Draft, Validation, Conceptualization.

## Declaration of competing interest

The authors declare that they have no known competing financial interests or personal relationships that could have appeared to influence the work reported in this paper.
