# Supplementary material for: Insect farming: A bioeconomy-based opportunity to revalorize plastic wastes
Source: Environ Sci Ecotechnol. 2024 Dec 24;23:100521. doi: 10.1016/j.ese.2024.100521 (PMC11758129; doi:10.1016/j.ese.2024.100521)
Supplement: Multimedia component 1 [file mmc1.docx]

**Supplementary Material**

Insect farming: a bioeconomy-based opportunity to revalorize plastic wastes

Juan C. Sanchez-Hernandez^1^ and Mallavarapu Megharaj^2^

^1^Laboratory of Ecotoxicology, Institute of Environmental Sciences, University of Castilla-La Mancha, 45071 Toledo, Spain.

^2^Global Centre for Environmental Remediation (GCER), College of Engineering, Science and Environment, University of Newcastle, Callaghan NSW 2308, Australia.

**Figure S1.** PRISMA flowchart and VOSviewer plot.

**Figure S2.** Bibliometric analysis of studies on insect-assisted biodegradation of plastics.

| A) | 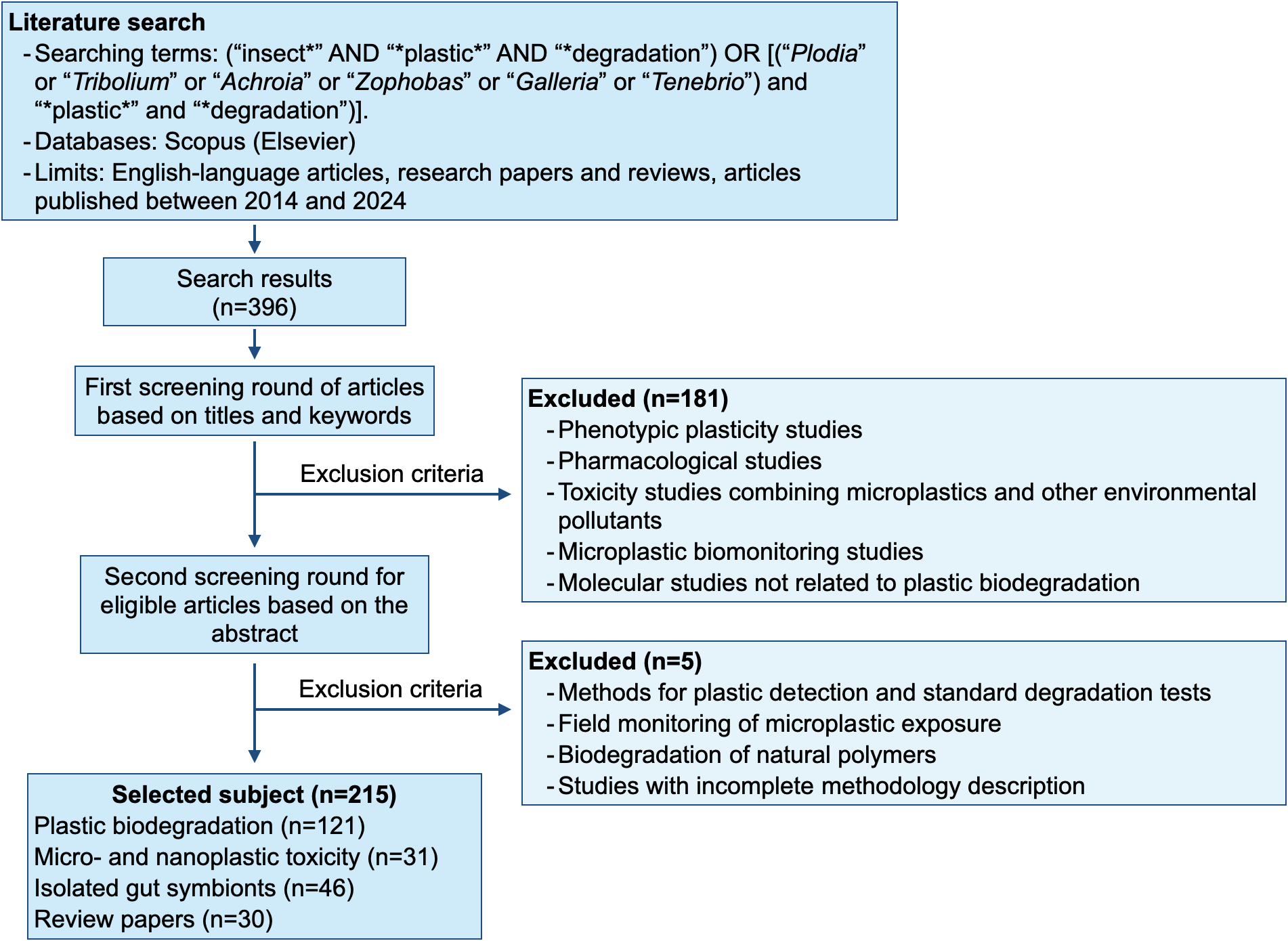 |
| --- | --- |
| B) | 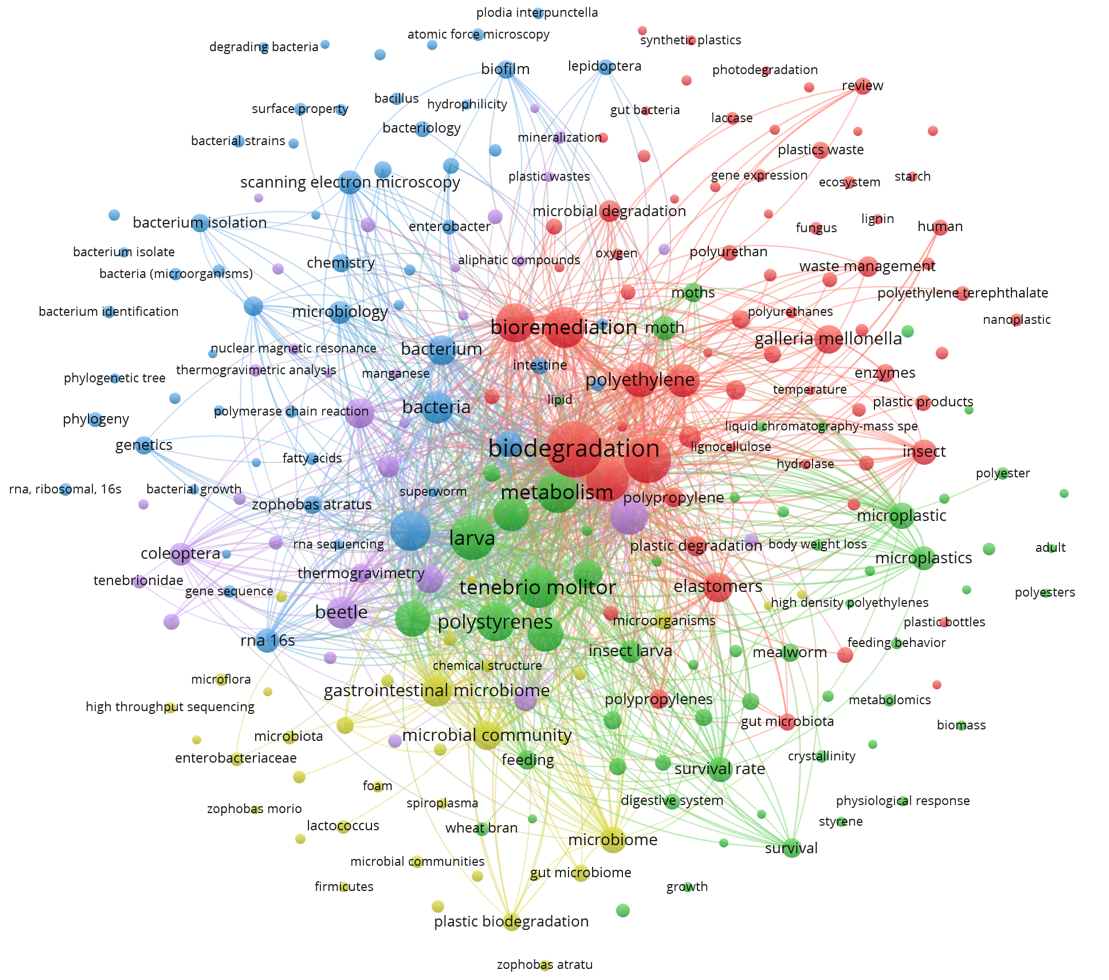 |

**Figure S1.** PRISMA flowchart (A) and VOSviewer plot (B). Relevant studies were retrieved from the electronic database Scopus^®^ (Elsevier) on November 9, 2024. We used a combination of taxonomic genera names in the searching terms as they represent key insect groups implicated in plastic degradation. The initial search yielded 396 articles, which were screened based on titles and abstracts to identify studies relevant to the objective of the Perspective. A total of 215 studies were selected and categorized into the following topics: (i) biodegradation of plastic polymers by insects, including the role of gut microbiota, (ii) micro- and nanoplastic toxicity in insects, (iii) isolation of insect gut symbionts with potential plastic degradation capabilities, and (iv) review articles.

|  |
| --- |

**Figure S2.** Bibliometric analysis of studies on insect-assisted biodegradation of plastics. A) Evolution of the cumulative number of published studies (research papers and reviews), countries significantly contributing in the topics (*Bio*=number of studies on plastic biodegradation by insects, including their gut symbionts; *Sym*=number of studies devoted on isolated microorganism from insect gut able to biodegrade plastic polymers in host-free media; *Tox*=number of studies addressing micro- and nanoplastic toxic effects on insect larvae exposed to plastic-rich diets). Note that some studies have involved more than one of these topics. B) Main taxonomic groups and synthetic polymer types involved in plastic biodegradation research.
